# Modulation of the expression of mimivirus-encoded translation-related genes in response to nutrient availability during *Acanthamoeba castellanii* infection

**DOI:** 10.3389/fmicb.2015.00539

**Published:** 2015-06-01

**Authors:** Lorena C. F. Silva, Gabriel M. F. Almeida, Felipe L. Assis, Jonas D. Albarnaz, Paulo V. M. Boratto, Fábio P. Dornas, Ketyllen R. Andrade, Bernard La Scola, Erna G. Kroon, Flávio G. da Fonseca, Jônatas S. Abrahão

**Affiliations:** ^1^Laboratório de Vírus, Departamento de Microbiologia, Instituto de Ciências Biológicas, Universidade Federal de Minas GeraisBelo Horizonte, Brazil; ^2^AQUACEN – Laboratório Nacional de Referencia para Doenças de Animais Aquáticos, Ministério da Pesca e Aquicultura, Universidade Federal de Minas GeraisBelo Horizonte, Brazil; ^3^URMITE CNRS UMR 6236 – IRD 3R198, Aix Marseille UniversitéMarseille, France; ^4^Laboratório de Virologia Básica e Aplicada, Departamento de Microbiologia, Instituto de Ciências Biológicas, Universidade Federal de Minas GeraisBelo Horizonte, Brazil

**Keywords:** mimivirus, tRNA, aminoacyl-tRNA-synthetases, translation, gene expression

## Abstract

The complexity of giant virus genomes is intriguing, especially the presence of genes encoding components of the protein translation machinery such as transfer RNAs and aminoacyl-tRNA-synthetases; these features are uncommon among other viruses. Although orthologs of these genes are codified by their hosts, one can hypothesize that having these translation-related genes might represent a gain of fitness during infection. Therefore, the aim of this study was to evaluate the expression of translation-related genes by mimivirus during infection of *Acanthamoeba castellanii* under different nutritional conditions. *In silico* analysis of amino acid usage revealed remarkable differences between the mimivirus isolates and the *A. castellanii* host. Relative expression analysis by quantitative PCR revealed that mimivirus was able to modulate the expression of eight viral translation-related genes according to the amoebal growth condition, with a higher induction of gene expression under starvation. Some mimivirus isolates presented differences in translation-related gene expression; notably, polymorphisms in the promoter regions correlated with these differences. Two mimivirus isolates did not encode the tryptophanyl-tRNA in their genomes, which may be linked with low conservation pressure based on amino acid usage analysis. Taken together, our data suggest that mimivirus can modulate the expression of translation-related genes in response to nutrient availability in the host cell, allowing the mimivirus to adapt to different hosts growing under different nutritional conditions.

## Introduction

*Acanthamoeba polyphaga mimivirus* (APMV) was the first discovered representative of the *Mimiviridae* family of amoeba-associated microorganisms involved with pneumonia (La Scola et al., 2003). Mature APMV particles are 700 nm in diameter and contain a double-stranded DNA genome of approximately 1.2 Mb that encodes approximately 1000 proteins, thereby surpassing the coding capacity of some bacteria (i.e., mycoplasma) (La Scola et al., 2003; Raoult et al., 2004; Moreira and Brochier-Armanet, 2008). The function of many of the ORFs encoded by APMV remain unknown. Some of these ORFs have never or rarely been found in other viruses, particularly components of the protein translation machinery (hereafter referred to as translation-related genes) including transfer RNAs (tRNAs), aminoacyl-tRNA-synthetases (aaRS), initiation factors, elongation factors, and release factors (Saini and Fischer, 2007; Claverie and Abergel, 2010; Legendre et al., 2010; Colson et al., 2011).

The translation of messenger RNA (mRNA) into protein in cellular organisms occurs through a complex process in the cytoplasm and consists of three main stages: initiation, elongation, and termination. Several players participate in this process, such as the ribosomes, tRNAs, and a varied enzymatic apparatus. In this context, aaRS are essential for the promotion of the correct interaction between tRNAs with their cognate amino acids. This reaction is called aminoacylation and leads to the formation of covalent bonds between the amino acid and the tRNA; once charged, the complex recognizes the respective codon in the mRNA and promotes the translation of the genetic information into a polypeptide chain (Ibba and Söll, 2004; Walsh and Mohr, 2011).

Cellular genes encoding the components of the protein translation machinery are regulated by different mechanisms. Some of the mechanisms involved in the regulation of aaRS expression are better characterized in bacteria and unicellular eukaryotes (Ryckelynck et al., 2005). The expression of aaRS in *Escherichia coli*, for example, is regulated in a manner that is dependent on the growth rate, but also by specific mechanisms induced in response to starvation of the cognate amino acid (Putzer and Laalami, 2000). In *Bacillus subtilis*, at least 16 aaRS genes are induced in response to starvation of their cognate amino acids via an uncharged tRNA-mediated transcription antitermination mechanism that appears to be conserved in Gram-positive bacteria (Putzer and Laalami, 2000; Ryckelynck et al., 2005). In budding yeast under amino acid starvation, stimulation of the translation of the transcriptional activator GCN4 in turn activates aaRS expression (Ryckelynck et al., 2005). This control is necessary to ensure the balance between the intracellular concentrations of uncharged and charged tRNAs to allow fine-tuning of not only translation, but also cellular metabolism as a whole in response to nutritional conditions in the extracellular environment. Various elements of specific amino acid biosynthetic pathways are involved in the regulation of aaRS expression and their disruption, either by raising or lowering the intracellular concentration of amino acids and other components important for the control of the expression and activity of these enzymes. Many different regulatory mechanisms allow both gene-specific control and global control of the expression of genes involved in protein translation (Neidhardt et al., 1975; Putzer and Laalami, 2000).

Although the existence of virally encoded translation-related genes is intriguing from a phylogenetic viewpoint, the functional relevance of such genes in the context of mimivirus infection is unknown. Here, we evaluated the expression of a number of mimivirus genes involved in translation during infection by APMV and other mimiviruses isolated in Brazil under different growth conditions. Our analysis revealed that mimiviruses were able to modulate the expression of viral translation-related genes according to the amoebal growth conditions during infection; for example, gene expression was increased under starvation conditions. We propose that the ability to adjust the expression of viral translation-related genes in response to nutrient availability may represent a remarkable example of viral adaptation to constantly changing conditions during infection of amoebal populations in the environment.

## Materials and Methods

### Virus Preparation and Cells

*Acanthamoeba polyphaga mimivirus*, a prototype of the *Mimiviridae* family, and APMV M4, a strain derived from APMV after 150 passages in amoeba culture (Boyer et al., 2011), were kindly provided by Dr. Didier Raoult (Aix Marseille Université, France). The Brazilian mimivirus isolates Kroon virus, Oyster virus, and Samba virus were produced and purified as previously described (La Scola et al., 2003). Briefly, *A. castellanii* (ATCC 30010) cells were grown in 75-cm^2^ cell culture flasks (Nunc, USA) in peptone-yeast extract-glucose (PYG) medium (Visvesvara and Balamuth, 1975) supplemented with 7% fetal calf serum (FCS, Cultilab, Brazil), 25 mg/mL fungizone (amphotericin B, Cristalia, Brazil), 500 U/mL penicillin, and 50 mg/mL gentamicin (Schering-Plough, Brazil). After reaching confluence, the amoebas were infected and incubated at 32°C until cytopathic effects were observed. Supernatants from the infected amoebas were collected and filtered through a 0.8-μm filter to remove cell debris. The viruses were purified by centrifugation through a sucrose cushion (24%), suspended in phosphate-buffered saline (PBS), and stored at -80°C. For the gene expression experiments, *A. castellanii* was maintained in PYG medium in the absence or in the presence of 7% FCS at 32°C or in Page’s amoeba saline (PAS) at 32°C to induce starvation.

### Infections and Experimental Design

To investigate the expression of mimivirus translation-related genes under distinct nutritional conditions, we selected eight genes based on the APMV genome: four tRNAs (leucyl, histidyl, cysteinyl, and tryptophanyl) and four aaRSs (methionyl, arginyl, tyrosyl, and cysteinyl). Twenty-four-well plates containing 1 × 10^5^ amoebas per well were infected with APMV, APMV M4, and the Brazilian isolates at a multiplicity of infection (MOI) of 10 and incubated at 32°C for 8 h, during which time the viral yield and expression of the selected genes are at their maxima (data not shown). We used three different amoebal growth conditions: PAS (a simple saline used for maintenance of the amoebas to induce starvation) and PYG (the growth medium commonly used to culture these cells under laboratory conditions), in the absence (PYG 0% FCS), and in the presence of FCS (PYG 7% FCS). The rationale behind this strategy is based on a nutritional scale of growth conditions: PAS < PYG 0% FCS < PYG 7 % FCS. Cells were collected and centrifuged, and the pellet was homogenized in 400 μL of PBS, from which 50 μL was used for titration in amoebas, while the remainder was pelleted again and used for total RNA extraction, reverse transcription, and quantitative PCR. The viral titer was determined using the TCID_50_ (tissue culture infective dose) method calculated with the Reed–Muench method (Reed and Muench, 1938). The titration was performed in 96-well Costar®microplates (Corning, NY, USA) containing 4 × 10^4^ amoebas per well in 100 μL of PYG with 7% FCS. Samples were serially diluted in PBS ranging from 10^-1^ to 10^-11^, and 100 μl of each dilution was inoculated onto amoebas (four wells per dilution, 200 μl final volume). Plates were incubated for 4 days at 32°C to determine the highest dilution that led to amoeba lysis (TCID_50_/ml). The results shown are representative of two independent experiments performed in duplicate.

### RNA Extraction, Reverse Transcription, and Real-Time PCR

Total RNA was extracted using the RNeasy kit (Qiagen, Germany). Reverse transcription was performed using the MMLV reverse transcriptase (Promega, USA) as recommended by the manufacturers. cDNA was used to determine the levels of tRNA and aaRS mRNA by quantitative PCR using specific primers (**Table 1**), SYBR Green Master Mix (Applied Biosystem, USA) and water in 10 μL reactions. Reactions were performed in the StepOne instrument (Applied Biosystem, USA). All reactions were previously optimized and presented high efficiency values. Relative gene expression analyses were performed using the ΔΔCt method and normalized to the expression of 18S ribosomal RNA (18S rDNA) and the viral RNA helicase mRNA.

**Table 1:** Primers used for quantitative PCR.

Gene	Forward primer	Reverse primer
Leucyl-tRNA	GGGATTCGAACCCACGACAT	ATAAGCAAAGGTGGCGGAGT
Histidyl-tRNA	TTAGTGGTAGAACTACTGTTTGTGG	TTTTCAAAAATGACCCGTACAGGAA
Cysteinyl-tRNA	ACAGTCAACTGGATCGTTAGC	AGGATCGTATCAGAATTGAACTGA
Tryptophanyl-tRNA	GTGCAACAATAGACCTGTTAGTTTA	ACCGGAATCGAACCAGTATCA
Methionyl tRNA synthetase	TGATTGGCGTGAATGGCTGA	ACCAATCACACTAGCCGGAA
Arginyl tRNA synthetase	GTGGGTGATTGGGGAACTCA	TGATACGGTCTCCAATCGGG
Tyrosyl tRNA synthetase	TTTGGCAAACCAATCGGCAA	TGGTTTTGAACCTAGTGGTCGT
Cysteinyl tRNA synthetase	TGCCAACCAGGTACACCAAA	TGCTCTTTGGAAAGGTCGATCA
18S rDNA	TCCAATTTTCTGCCACCGAA	ATCATTACCCTAGTCCTCGCGC
Viral RNA helicase	ACCTGATCCACATCCCATAACTAAA	GGCCTCATCAACAAATGGTTTCT

### Amino Acid Usage

To investigate the profile of amino acid usage between mimivirus isolates and *A. castellanii*, the protein sequences predicted from the Brazilian mimivirus genomes [APMV (GenBank accession HQ336222.2) and APMV M4 (GenBank accession JN036606.1)] and amoebal sequences obtained from the NCBI GenBank amino acid sequence database were subjected to amino acid composition calculation using the program CGUA (General Codon Usage Analysis). The amino acid usages were expressed as percentages and reflected the contribution made by each amino acid. The amino acid usage of *A. castellenii* was calculated from the 45,664 protein sequences available in the NCBI database.

### tRNA and aaRS Gene Analysis

To investigate possible polymorphisms among translation-related gene sequences of the five mimivirus isolates analyzed, the tRNA and aaRS sequences of APMV (GenBank accession HQ336222.2), APMV M4 (GenBank accession JN036606.1), Kroon virus, Oyster virus, and Samba virus were aligned with GenBank references using ClustalW and manually aligned using MEGA software version 5.2 (Arizona State University, Phoenix, AZ, USA). Whole genome sequences of Brazilian mimivirus isolates were obtained using an Illumina MiSeq instrument (Illumina Inc., San Diego, CA, USA) with the paired-end application (unpublished data). Brazilian mimiviruses genomes were searched for translation-related genes using available sequences from giant viruses available in GenBank. After the alignment, the sequences were thoroughly analyzed and compared for similarity, promoter sequences, gaps, and polyadenylation signals. The APMV ORF with no predicted promoter regions in GenBank (methionyl-RS and tryptophanyl-tRNA), had their promoters analyzed manually.

### Statistical Analyses

Statistical analyses were performed using GraphPad Prism software (San Diego, CA, USA). The significance analysis was performed by comparing the average of the obtained results within the groups. The values were subjected in different combinations to one-way ANOVA tests and Bonferroni post-tests (95% confidence intervals). Differences between groups were considered significant when the *p*-values were smaller than 0.05.

## Results

### Amino Acid Usage

**FIGURE 1:**
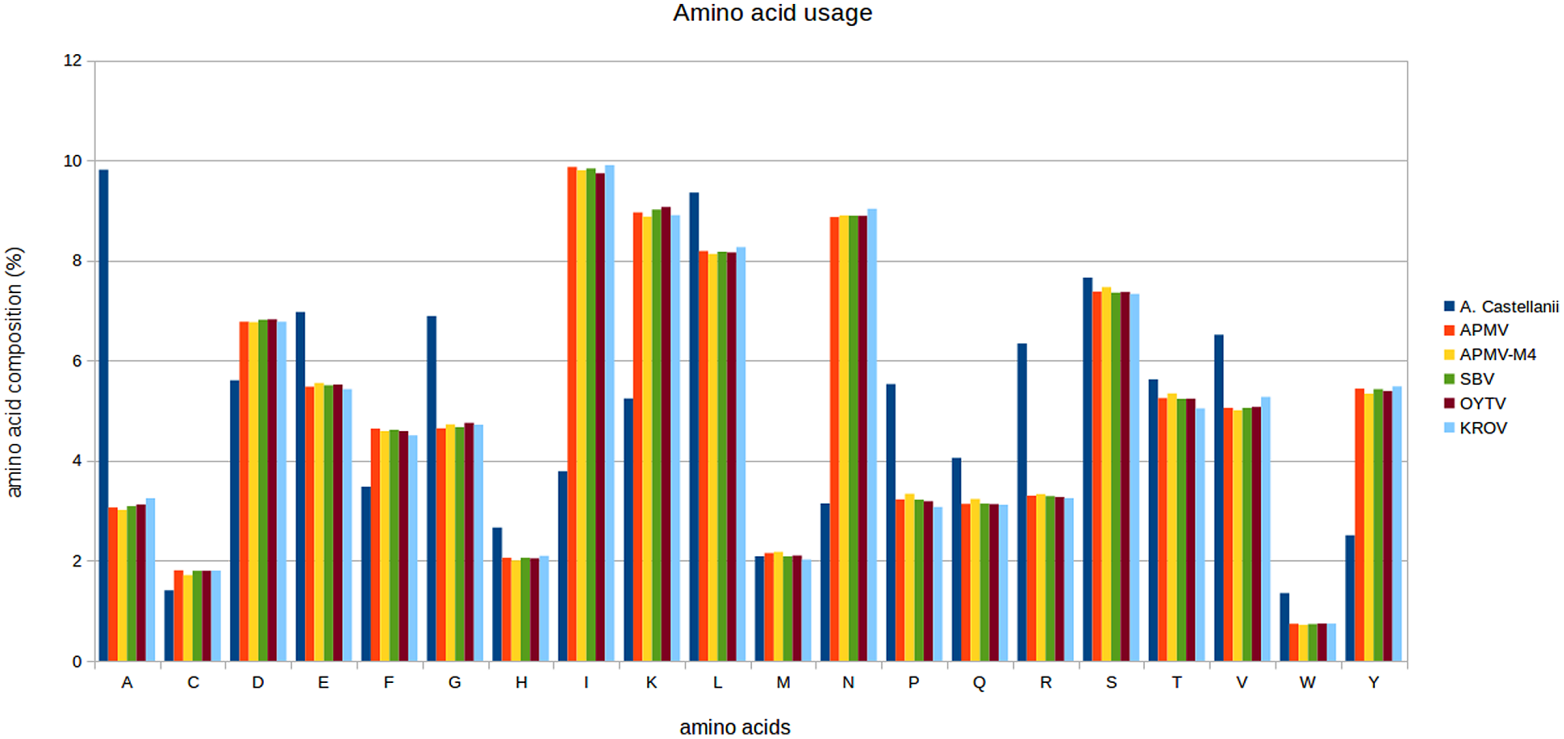
**Comparative amino acid usage analysis of *Acanthamoeba castellanii* and mimivirus strains**. The amino acid usage for protein sequences of Brazilian mimivirus strains, *A. polyphaga mimivirus* (APMV) and APMV M4 strains, and the *A. castellanii* amoeba were calculated using the CGUA (General Codon Usage Analysis) tool.

Our results clearly showed a different profile of amino acid usage between the *Acanthamoeba* and mimiviruses isolates (**Figure 1**). In contrast, the amino acid usage in APMV, APMV M4, and the other Brazilian mimivirus isolates was very similar (**Figure 1**). Two of the amino acids for which mimiviruses encode a cognate aaRS [cysteine (C) and methionine (M)] are less frequently found in both *A. castallanii* and the predicted proteome of the mimivirus isolates compared to arginine (R; most frequent in the host proteome) and tyrosine (Y; more frequently found in the viral proteome). The leucine, histidine, and tryptophan tRNAs were less frequently found in the viral predicted proteome compared to the host.

### Expression of tRNA and aaRS Genes

To evaluate the effect of nutrient availability on the expression profile of virally encoded tRNA and aaRS during infection with different mimivirus isolates (APMV, APMV M4, Kroon virus, Oyster virus, and Samba virus), cells infected under different growth conditions were collected, processed, and assayed by quantitative PCR. The quantitative PCR results were expressed as arbitrary units, fitted to standard curves generated for each target gene (Supplementary Figure S1), and normalized using the amoebal 18S rDNA gene levels.

**FIGURE 2:**
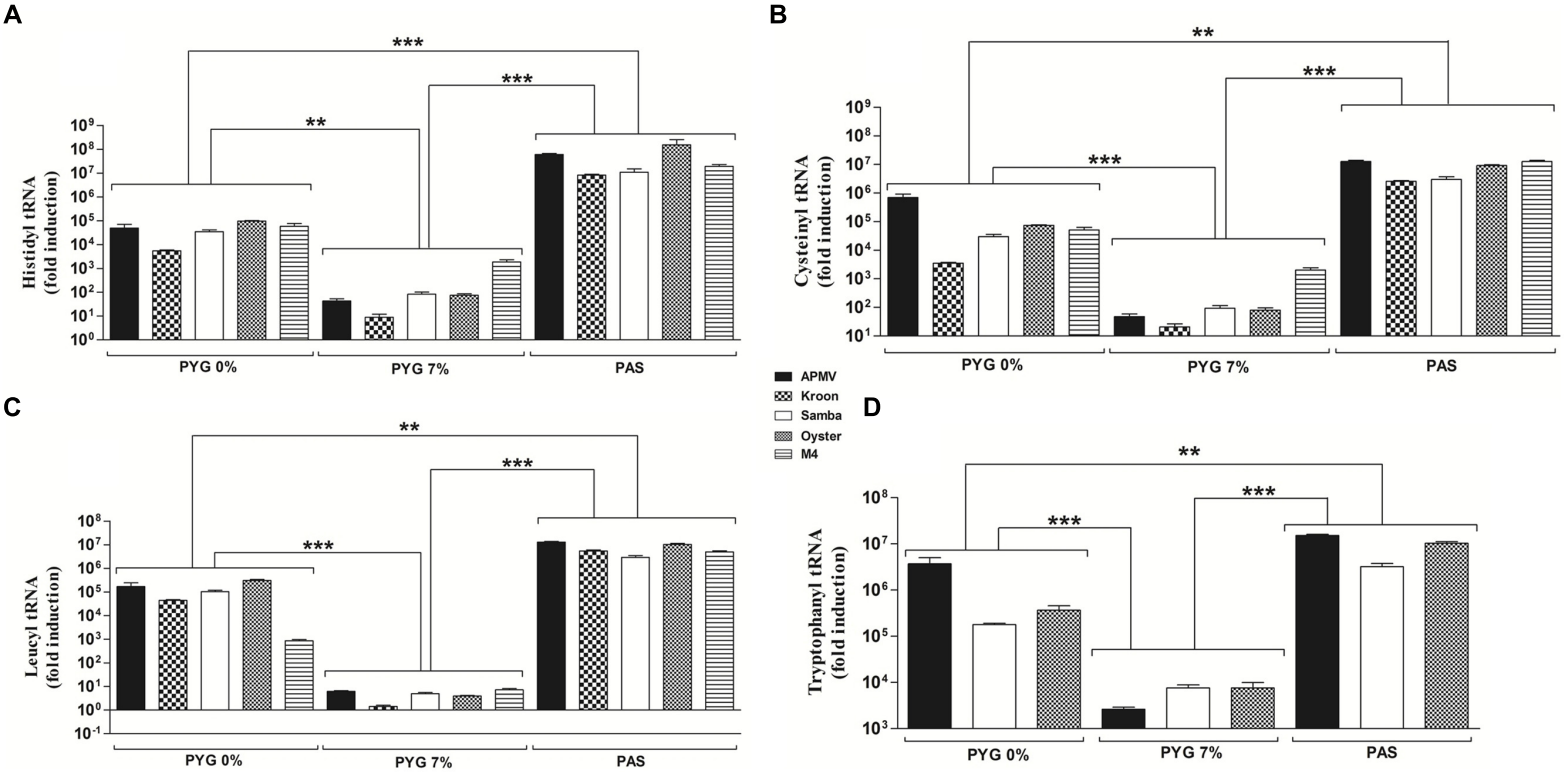
**tRNA expression by different mimivirus strains**. Cells of *A. castellanii* were plated in 24-well plates (1 × 10^5^ cells per well), infected for 8 h with different mimivirus strains in different culture medium, subjected to total RNA extraction, and reverse transcription and the resulting cDNA was used as a template for quantitative PCR. **(A)** Histidyl-tRNA. **(B)** Cysteinyl-tRNA. **(C)** Leucyl-tRNA. **(D)** Tryptophanyl-tRNA. The quantitative PCR results were expressed in arbitrary units, fitted to a standard curve, normalized to levels of the constitutive amoebal 18S rDNA gene and presented as the mean ± SD from a representative experiment conducted in duplicate. The values were subjected in different combinations to one-way ANOVA tests and Bonferroni post-tests (95% confidence intervals). Differences between groups were considered significant when the *p*-values were smaller than 0.05. ^∗∗^*p* < 0.01; ^∗∗∗^*p* < 0.001.

**FIGURE 3:**
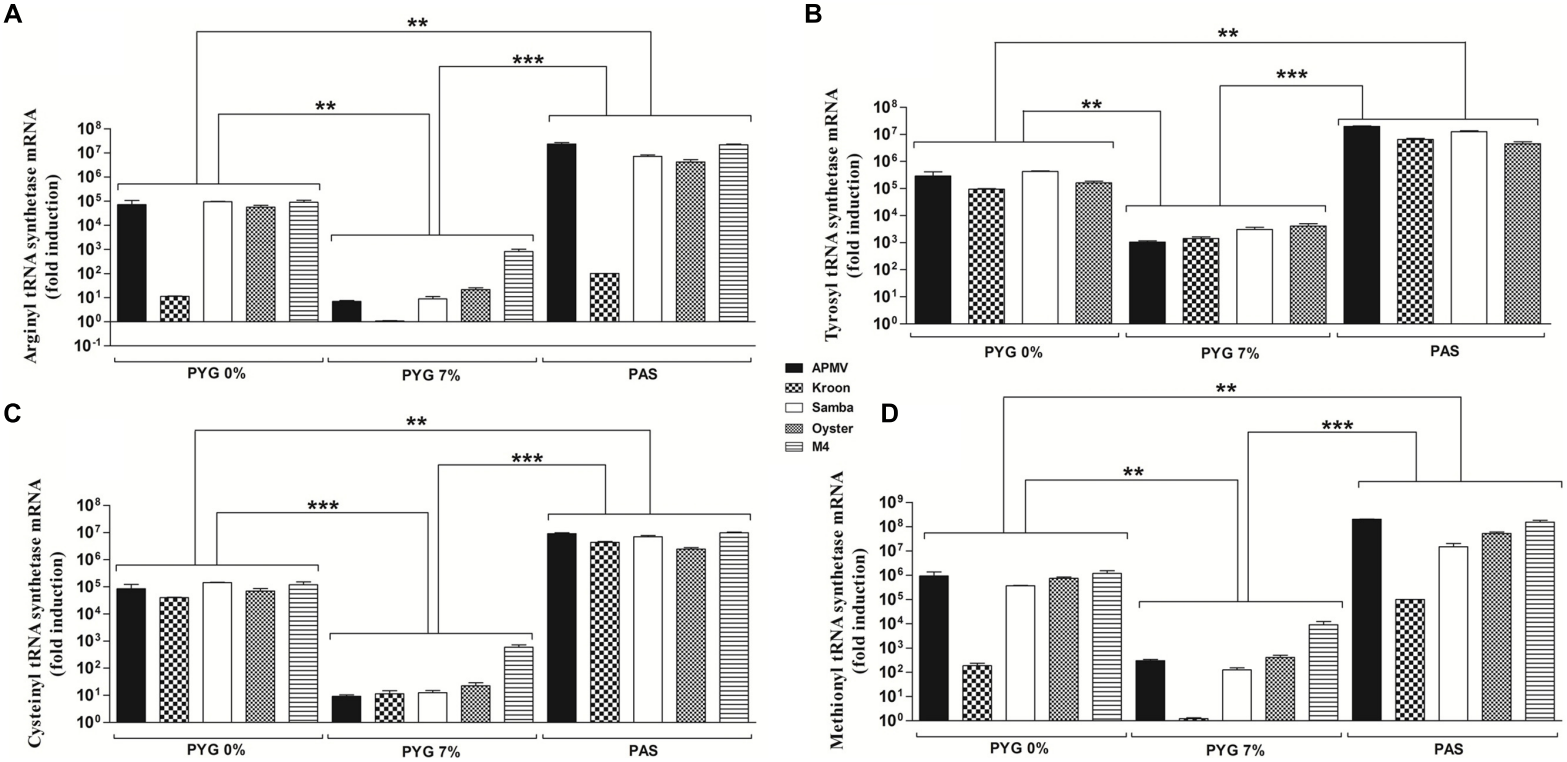
**Aminoacyl-tRNA-synthetase (aaRS), messenger RNA (mRNA) expression by different mimivirus strains**. Cells of *A. castellanii* were plated in 24-well plates (1 × 10^5^ cells per well), infected for 8 h with different mimivirus strains in different culture medium, subjected to total RNA extraction and reverse transcription and the resulting cDNA used as a template for quantitative PCR. **(A)** Arginyl-RS mRNA levels. **(B)** Tyrosyl-RS mRNA levels. **(C)** Cysteinyl-RS mRNA. **(D)** Methionyl-RS mRNA levels. The real-time PCR results were expressed arbitrary units, fitted to a standard curve, corrected using normalization with amoebal 18S rDNA gene expression levels and presented as the mean ± SD from a representative experiment conducted in duplicate. The values were subjected in different combinations to one-way ANOVA tests and Bonferroni post-tests (95% confidence intervals). Differences between groups were considered significant when the *p*-values were smaller than 0.05. ^∗∗^*p* < 0.01; ^∗∗∗^*p* < 0.001.

Our results revealed that viral tRNA and aaRS mRNA expression varied according to the viral strain and growth medium used (PAS, PYG 0% FCS, or PYG 7% FCS; **Figures 2** and **3**). Overall, mimivirus-encoded tRNA and aaRS gene expression was significantly lower in cells infected under high nutrient availability conditions (PYG 7% FCS) in comparison to cells infected under PYG 0% FCS or starvation (PAS) conditions. Viruses infecting amoeba maintained in PAS medium presented the highest viral mRNA expression for all analyzed genes (*p* < 0.001 or *p* < 0.01; **Figures 2** and **3**). The different mimivirus isolates showed some variation in translation-related gene expression profiles between them when infection was performed under the same conditions. For example, there was a distinctive expression profile was observed for Kroon virus-encoded cysteinyl-tRNA in PYG 0% FCS and arginyl- and methionyl-RS under all amoebal growth conditions (**Figures 2** and **3**). Neither Kroon virus nor M4 presented detectable levels of tryptophanyl-tRNA due to the absence of this gene in both isolates, as revealed by genomic prediction (Supplementary Figure S2). Similarly, M4 does not encode (or express the mRNA) of tyrosyl-RS. We also performed a quantification of the viral RNA helicase gene by real-time PCR. The results revealed that viruses and nutritional conditions did not significantly influence RNA helicase expression, suggesting that the effect observed for the tRNA, and aaRS mRNA expression levels cannot be generalized for all genes under starvation conditions (Supplementary Figure S3).

**FIGURE 4:**
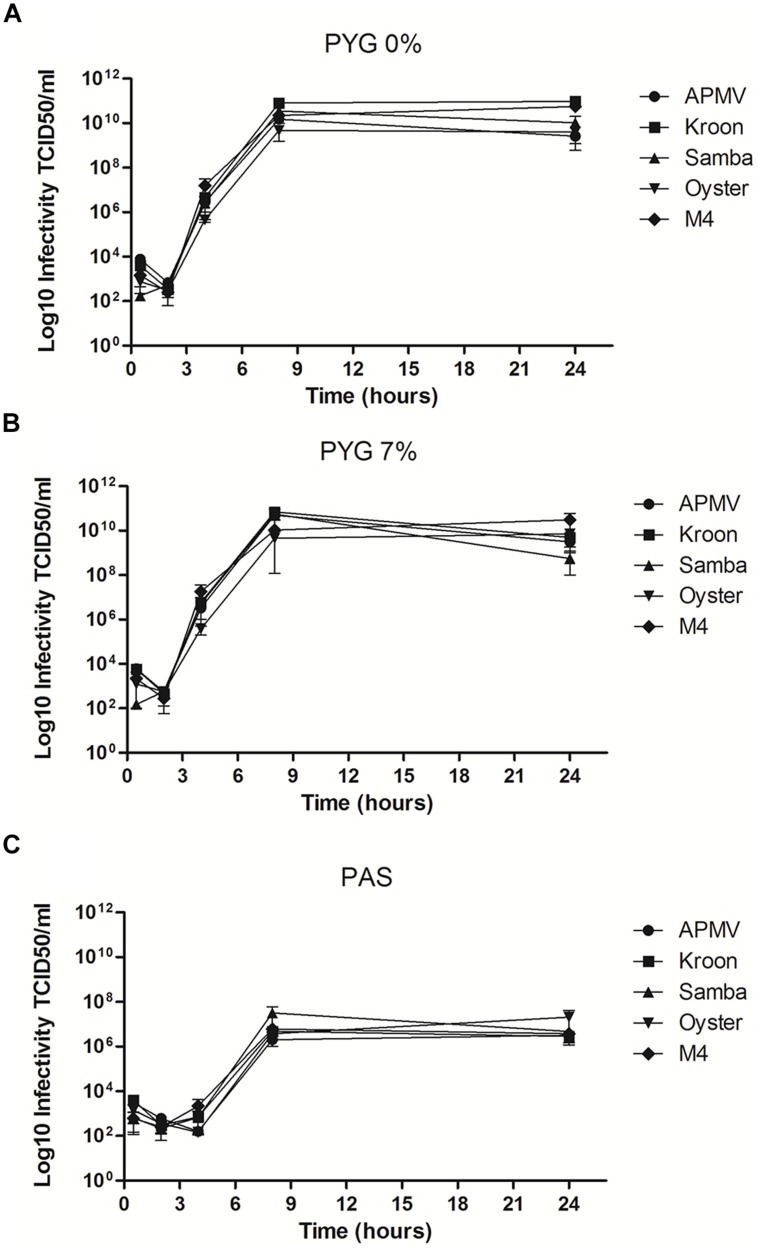
**Replication of different mimivirus strains in *A. castellanii*.** Cells of *A. castellanii* were plated in 24-well plates (1 × 10^5^ cells per well) and infected with APMV, APMV M4, Kroon virus, Oyster virus, and Samba virus at an multiplicity of infection (MOI) of 10 for the 8 h in three different culture media: **(A)** peptone-yeast extract-glucose (PYG) 0% FCS; **(B)** PYG 7% FCS and **(C)** Page’s amoeba saline (PAS). The infectivity of viral particles was evaluated by titration in amoebas by TCID_50_. The results are the mean ± SD of a representative experiment conducted in duplicate.

We also performed one-step growth curves. Our results showed that the five evaluated mimivirus strains were able to productively infect the amoebas under the three tested growth conditions (**Figure 4**). All five isolates showed a substantial increase in viral yield at 4 h post-infection (h.p.i.), with a peak at approximately 8 h.p.i. that was maintained until 24 h.p.i. (**Figure 4**). There were no significant differences between the growth curves of the tested mimivirus isolates when the infection was performed in the presence of PYG 0% FCS or PYG 7% FCS (**Figures 4A,B**). In contrast, the viral yield was approximately 1,000-fold lower under starvation conditions (PAS solution; **Figure 4C**). Although the mimivirus isolates showed a lower progeny yield in the starved amoebas, the low nutrient availability induced higher expression of the virally encoded translation-related genes compared to PYG with 0 or 7% FCS.

### Polymorphisms in Mimivirus-Encoded Translation-Related Genes

To evaluate whether the differences in translation-related gene expression during infection by the different mimivirus isolates could be associated with polymorphisms in the genes, we analyzed the sequences of the eight translation-related genes investigated in this study using a nucleotide alignment constructed with the MEGA software version 5.2. The fully sequenced and annotated APMV genome (the prototype of the *Mimiviridae* family) showed the presence of tRNAs, aaRSs, and elongation factors among other elements involved in the process of protein synthesis. Thus, this virus was selected for analysis in this work and served as the source for primer design and comparison with the sequences of the other isolates studied. These comparisons showed that the APMV and Samba virus orthologs of the investigated genes under study exhibited 100% similarity. Six of the genes in APMV and M4 exhibited 100% similarity, with the exception of tyrosyl-RS and tryptophanyl-tRNA that were reported to have been deleted during the successive passages of APMV that gave rise to the M4 strain. Several polymorphisms were detected during the comparison of the translation-related genes encoded by APMV and two other Brazilian mimivirus strains (Kroon virus and Oyster virus; Supplementary Figure S2). Among the studied Brazilian isolates, Kroon virus presented the most remarkable polymorphisms, including the absence of the tryptophanyl-tRNA coding region (Supplementary Figure S2). Moreover, careful analysis of the nucleotide sequences of the translation-related genes encoded by Oyster virus and Kroon virus revealed several differences in the promoter regions and polyadenylation signals, with important nucleotide changes in genes such as the cysteinyl-tRNA and methionyl- and arginyl-RS due to the presence of gaps in these regions (Supplementary Figures S2 and S4).

## Discussion

Not enough all the structural complexity presented by giant viruses, in recent years, studies showed that they also have large and complex genomes (La Scola et al., 2003; Raoult et al., 2004; Moreira and Brochier-Armanet, 2008). The fact that these viruses possess genes that encode proteins involved in protein translation is highly intriguing, but the biological essentiality and importance of these genes remains unknown (Jeudy et al., 2012; Colson et al., 2013). In this context, our study presents new data concerning the giant viruses and their translation-related genes.

Viruses are known to rely exclusively on the host cell protein synthesis machinery for the translation of viral proteins. Therefore, the discovery that giant viruses encoded translation-related genes in their genomes was quite surprising and led virologists to question the boundaries between giant viruses and cellular organisms (Raoult et al., 2004; Moreira and Brochier-Armanet, 2008). The prototype of *Mimiviridae* family, APMV, encodes six tRNAs (histidyl, cysteinyl, tryptophanyl, and leucyl, the latter of which appears in three copies in the genome) and four aaRSs (methionyl, arginyl, tyrosyl, and cysteinyl) (Colson et al., 2013). We propose that the existence of translation-related genes encoded in the mimivirus genome suggests that they may exert some control on the cellular translational machinery in a way that cannot be performed by their cellular counterparts and is necessary for successful viral replication.

Evaluation of amino acid usage of the five mimivirus strains used in this study and *A. castellanii* suggested that the amino acid usage was very similar between viral strains but differed from *A. castellanii* (**Figure 1**), as previously suggested by Colson et al. (2013). Considering the tRNA and aaRS elements analyzed here, we hypothesize that the most used amino acids [i.e., leucine (L) and tyrosine (Y)] are important for the viral machinery. Thus, all of the analyzed viral isolates possess a leucyl-tRNA gene. Arginine (R) and methionine (M) are less used, but can be important because R is widely used by *A. castellanii*, while M is the initiating amino acid. Histidine (H) and cysteine (C) are also maintained in the viral machinery although they are used less (even by *A. castellanii*), but can be important to the process of infection of other hosts, including *Homo sapiens* (data not shown). Finally, the use of tryptophan (W) is not frequent, which accounts for the low pressure of maintenance of the viral machinery demonstrated by the lack of tryptophanyl-tRNA in Kroon virus and APMV M4.

Relative expression analysis revealed that mimivirus is able to modulate the expression of translational-related genes according to nutrient availability during amoebal infection (**Figures 2** and **3**). Under starvation conditions, mimivirus-encoded aaRS mRNA expression resembles the induction of cellular aaRS expression under the same growth condition, as previously observed for bacteria and *Saccharomyces cerevisae* (Ryckelynck et al., 2005). In the yeast, low amino acid availability is sensed by the GCN2 protein kinase that is activated by uncharged tRNAs. GCN2 phosphorylates the eIF2 initiation factor, inhibiting its function in translation initiation. Inactivation of eIF2 leads to translation of GCN4, which in turn is a transcriptional activator that activates the transcription of aaRS by binding to the aaRS gene promoters (Ryckelynck et al., 2005). Whether this amino acid availability sensor operates in *A. castellanii* awaits investigation. Furthermore, there is the possibility of an interplay between nutrient availability sensing of the host amoeba and the stimulation of the mimivirus tRNA and aaRS genes during infection under starvation conditions. Considering the reduced viral growth under starvation (as shown by the one-step-growth curve experiments in **Figure 4C**), we can hypothesize that circumventing limited nutrient availability is critical for successful mimivirus propagation in natural amoebal populations.

Some isolates presented differences in gene expression in comparison to other isolates. Genetic differences between isolates were discovered when the nucleotide and predicted amino acid sequences of translation-related genes were analyzed, revealing polymorphisms in gene promoter regions that could explain this phenomenon. Polymorphisms were detected only in Oyster virus and with high frequency in the Kroon virus-encoded translation-related genes and regulatory elements (Supplementary Figure S2). A careful analysis of these sequences from these two Brazilian isolates (especially Kroon virus) demonstrated nucleotide substitutions in the promoter regions and polyadenylation signals of some genes and the absence of these regulatory sequences in other genes, including leucyl-tRNA, cysteinyl-RS, cysteinyl-tRNA, methionyl-RS, and arginyl-RS (Supplementary Figure S2). For the arginyl-RS gene that presented the lowest expression levels during infection by Kroon virus, we observed an approximately 15% difference in the nucleotide sequence between the early promoter and the initiation codon and an approximately 8% difference in the region containing the late promoter. Furthermore, approximately 46 bp are missing in the 3′ end of the Kroon virus-encoded arginyl-RS compared to the APMV ortholog (Supplementary Figure S4). We propose that these differences may help explain the different levels of translation-related gene expression in Kroon virus compared to the other mimivirus strains (Figures 2 and 3 and Supplementary Figure S2). APMV M4, which was obtained after successive passages of APMV under allopatric conditions, lost several ORFs including those encoding the tryptophanyl-tRNA and tyrosyl-RS, suggesting that these ORFs were not essential under these culture conditions. Therefore, these genes are likely important in sympatric growth conditions, and APMV would be able to compete better with other microorganisms associated with amoebas by encoding these ORFs and expressing them according to nutrient availability (Boyer et al., 2011; Colson and Raoult, 2012).

The tRNA and aaRS present in giant virus genomes are not pseudogenes, because they are temporally expressed (early, intermediate, and late) during the replication cycle and they are different from cellular genes. Some of these genes are expressed at high levels at different times of infection, suggesting that they are involved in protein translation from the beginning to the late phases of the cycle (Legendre et al., 2010). Moreover, two mimivirus-encoded aaRS (methionyl-RS and tyrosyl-RS) possess genuine enzymatic activity (Abergel et al., 2007; Legendre et al., 2010). Therefore, considering the fact that these viral proteins are differentially expressed depending on the mimivirus isolate and the nutrient availability during infection, we can speculate that genetic and biological differences among viral strains probably reflect their natural history in the environment and their ability to adapt to different hosts under different environmental conditions. In conclusion, our study showed that translation-related genes encoded by giant viruses are differentially expressed in response to nutrient availability during infection of the amoebal host. This finding raised three key questions that need further investigation to be answered: (i) how the regulation of viral tRNA and aaRS gene expression is coordinated with the host cell response to low nutrient availability, especially amino acid starvation; (ii) the functional relevance of these viral translation-related gene products for productive viral infection and for host range adaptation in natural populations of susceptible hosts; and (iii) the significance of the polymorphisms in the regulatory and coding regions of some Brazilian mimivirus isolates for the expression and function of the viral translation-related gene products.

## Conflict of Interest Statement

The authors declare that the research was conducted in the absence of any commercial or financial relationships that could be construed as a potential conflict of interest.
