# Supplementary figures and images for: Modulation of the expression of mimivirus-encoded translation-related genes in response to nutrient availability during Acanthamoeba castellanii infection
Source: Front Microbiol. 2015 Jun 1;6:539. doi: 10.3389/fmicb.2015.00539 (PMC4450173; doi:10.3389/fmicb.2015.00539)

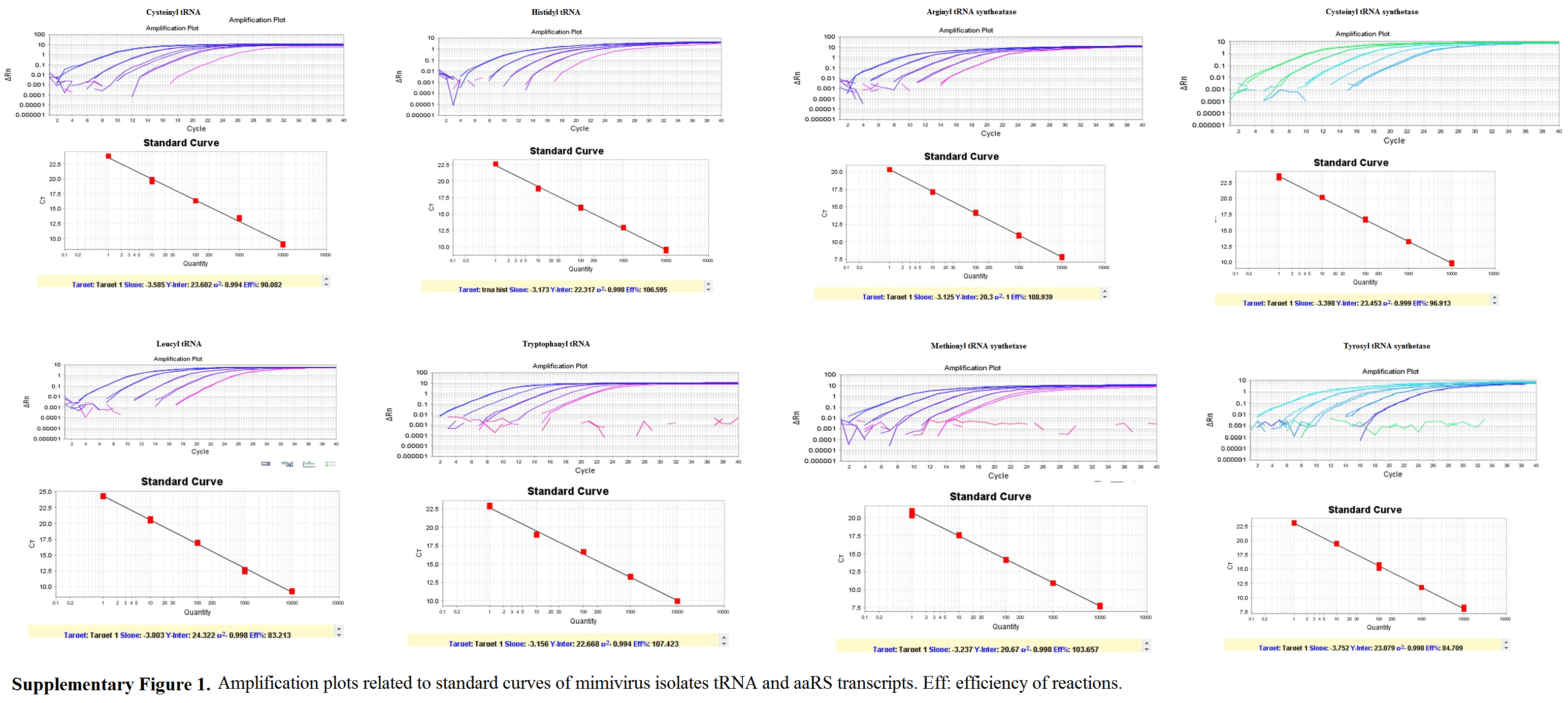

Supplement: Supplementary file 1 [file Image_1.TIF]

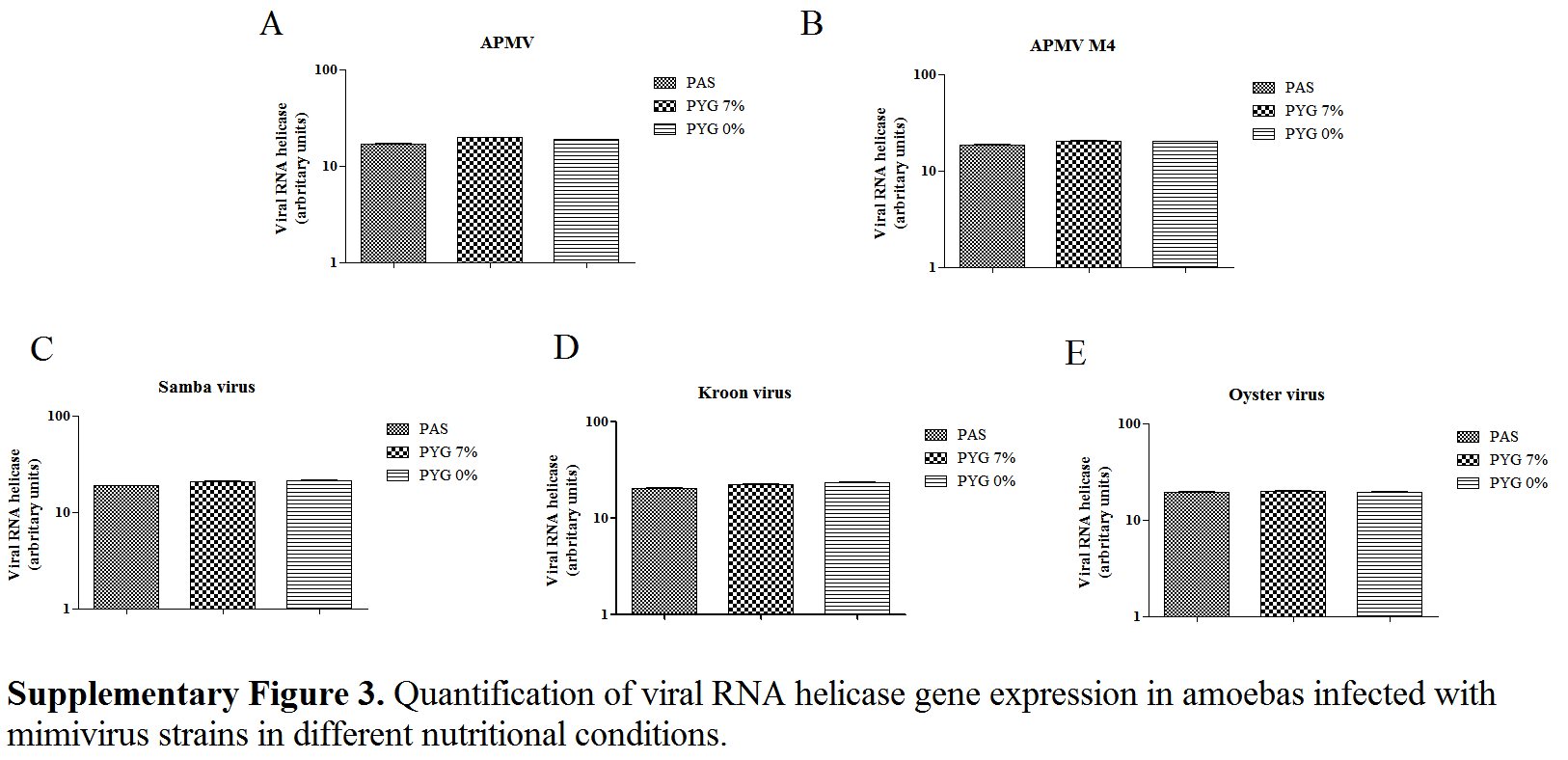

Supplement: Supplementary file 3 [file Image_3.TIF]

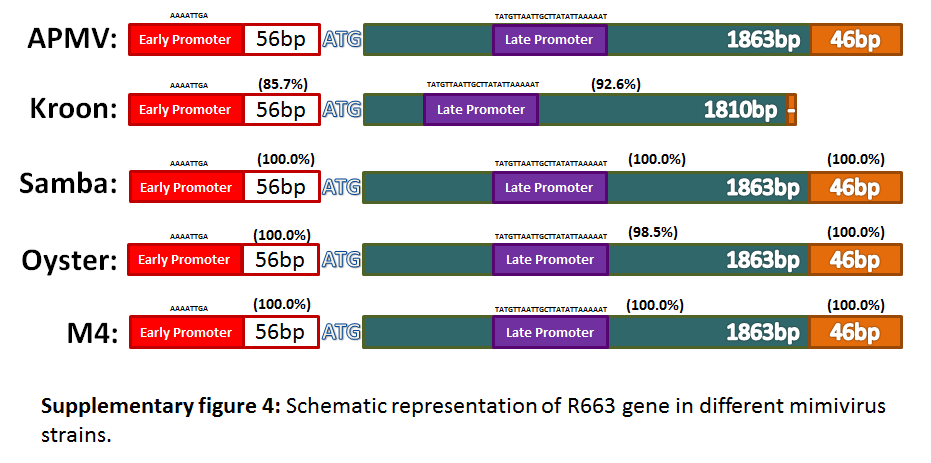

Supplement: Supplementary file 4 [file Image_4.TIF]
