# Supplementary material for: Modulation of the expression of mimivirus-encoded translation-related genes in response to nutrient availability during Acanthamoeba castellanii infection
Source: Front Microbiol. 2015 Jun 1;6:539. doi: 10.3389/fmicb.2015.00539 (PMC4450173; doi:10.3389/fmicb.2015.00539)
Supplement: Supplementary file 2 [file Image_2.PDF]

Supplementary Figure 2. tRNA and aaRS genes analysis.

X Early promoter

X late promoter

X Gene

X Polyadenylation signal

Histidyl tRNA

>APMV/SAMBA VIRUS/APMV M4 (100%)
TCAATTTTTTCAA**ATG**CGTTTGAAAATAAATTAAAA**ATG**TATAATTTATTATTGTGGGTAAAACCACGGATTTCCAAAAATTCAACAATACTATAAGATCCGTTAGTTTAGTGGTAGAACTACTGTTTGTGGGACGGTCGACACA
GGTTCGATTCCTGTACGGGTCATTTTTGAAAATTTTGAAATCCAGTTGATTGTAGATTTACAGTCAACTGGAT

>KROON VIRUS (96% ID, 0% gap)
TCAATTTTTTTTAA**ATG**CGTTTGAAAATAAATTAAAAATGTATAATCTATTTTTGTGGGTAAAACCACGGATTTCCAAAAATCCAACAATACTATAAGATCCGTTAGTTTAGTGGTAGAACTACTGTTTGTGGGACGGTCGACAC
AGGTTCGATTCCTGTACGGGTCATCGT-GAAAATTTTGAAATCCAGTTGATTGTAGATTTACAGTCAACTGGAT

>OY (98%ID, 0% gap)
TCAATTTTTTTAA**ATG**CGTTTGAAAATAAATTAAAA**ATG**TATAATTTATTATTGTGGATAAAACCCACGGATTTCCAAAAATTCAACAGTATTATAAGATCCGTTAGTTTAGTGGTAGAACTACTGTTTGTGGGACGGTCGACAC
AGGTTCGATTCCTGTACGGGTCATTTTTGAAAATTTTGAAATCCAGTTGATTGTAGATTTACAGTCAACTGGAT

Leucyl tRNA

>APMV/SAMBA VIRUS/APMV M4 (100%)
TCAATTTTAATTGATACATTTATA**ATG**ATTCAA**ATG**AATCATTATAAATAGTGTGA**ATG**CAAAGGGTGGGATTCGAACCCACGACATCAAAAGATAGTAGATCTTAAGTCTACCGCGTTAGACCACTCCGCCACCTTTGCTTATT
TAATTAATATTAAATTATTTTTATATCATTTTGATATTTTGATATCTTGATAATTGTTGGATTGGTAATTTTTAATTGATAAAAAATTACACAATTAAAAATTAC

>KROON VIRUS (90% ID, 4% gap)
TCAATTTTAATTGATACATTTATAATGATTCAATCGAATAATTATAAATAATGTGA**ATG**CAAAGGGTGGGATTCGAACCCACGATATCAAATGATACTAGATCTTAAGTCCAGCGCGTTAGACCACTCCGCCACCTTTGCTTATT
TTAATGTAATTAATTTCTTTTTATATCATTTTTATATTTTGATA--------ATTGTTGGATTGGTAATTTTTAATTGATAAAAAATTATACA-TTAAAAATTAC

>OYSTER VIRUS (93% ID, 1% gap)

TCAATTTTAATTGATACATTTATA**ATG**ATTCAA**ATG**AATCATTATAAATAGTGTGAATGCAAAGGGTGGGATTCGAACCCACGACATCAAAAGATAGTAGATCTTAAGTCTACCGCGTTAGACCACTCCGCCACCTTTGCTTATT
TTAATGTAATTAATTTTCTTTTTATATCATTTTGAAATTTTGATAATTTGATAATTGCCAAATTGGTAATTTTTAATTGATAAAAAATTTATACACTAAAAATTAC

Cysteinyl tRNA

>APMV/SAMBA VIRUS/APMV M4 (100%)

TCAATTTTTTCAAATGCGTTTGAAAATAAATTAAAAATGTATAATTTATTATTGTGGGTAAAACCACGGATTTCCAAAAATTCAACAATACTATAAGATCCGTTAGTTTAGTGGTAGAACTACTGTTTGTGGGACGGTCGACACA
GGTTCGATTCCTGTACGGGTCATTTTTGAAAATTTTGAAATCCAGTTGATTGTAGATTTACAGTCAACTGGATCGTTAGCTCA**ATG**GTAGAGCGCTCGCCTGCAGAGCGATAGGTTATCAGTTCAATTCTGATACGATCCTTTTT
AGATGTATAAATTTATGCATCTAAAAA

>KROON VIRUS (96% ID, 1% gap)

TTGAAAATAAATTAAAAATGTATAATCTATTTTTGTGGGTAAAACCACGGATTTCCAAAAATCCAACAATACTATAAGATCCGTTAGTTTAGTGGTAGAACTACTGTTTGTGGGACGGTCGACACAGGTTCGATTCCTGTACGGG
TCATCGT-
GAAAATTTTGAAATCCAGTTGATTGTAGATTTACAGTCAACTGGATCGTTAGCTCA**ATG**GTAGAGCGCTCGCCTGCAGAGCGATAGGTTATCAGTATCAATTCTGATACGATCCTTTTTTAGATGTATATTTTTATGCATCTAAA
AA

>OYSTER VIRUS (98% ID, 0% gap)

TCAATTTTTTTAAATGCGTTTGAAAATAAATTAAAAATGTATAATTTATTATTGTGGATAAAACCCACGGATTTCCAAAAATTCAACAGTATTATAAGATCCGTTAGTTTAGTGGTAGAACTACTGTTTGTGGGACGGTCGACAC
AGGTTCGATTCCTGTACGGGTCATTTTTGAAAATTTTGAAATCCAGTTGATTGTAGATTTACAGTCAACTGGATCGTTAGCTCA**ATG**GTAGAGCGCTCGCCTGCAGAGCGATAGGTTATCAGTTCAATTCTGATACGATCCTTTT
TTAGATGTATAAATTTATGCATTTAAAAA

Tryptophanyl tRNA

>APVMV/SAMBA VIRUS (100%)

TCCATAAAACCACAATCTAAATATCACAGACAAAAAAAAATTTAGAAATAATTTTTCATCACAAAATAAATTTCACCATCAAAAAATTTTTCCTCGACAAAAATTCTCTAAATTTCACAGATAAAAATGGGTGGGAATTATATAAA
AATTTTTTCTTTACTGTAAGTGTAAAGATCTGTTGTTGCATTTAGTGCAACAATAGACCTGTTAGTTTA**ATG**GTAAAACGGTAGCCTCCAGAGTGATTGATACTGGTTCGATTCCGGTATAGGTCCATTTAATAAAAAAAAACAAT
TAATTAGTATTAGTTGTTTTTTT

APMV M4(deleted)

KROON VIRUS (deleted)

OYSTER VIRUS (ID 96%, 0% gap)

TCCATAAAACCACAATCTAAATATCACAGACAAAAAAAATTTAGAAATAGTTTTTCATCACAAAATAAATTTCACCATCAAAAAATTTTTCCTCGACAAAAATTCTCTAAATTTCATAGATAATAATGGTTGGAAATTATATAAA
AATTTTTTCTTTACTGTAAGTGTATAGATCTGTTGCTGCATTTAGTGCAACAATAGACCTGTTAGTTTAATGGTAAAACGGTCGCCTCCAGAGTGATTGATACTGGTTCGATTCCGGTACATGTCCTTTTAATAAAAAAAACAAT
TAATTAGTATTAGTTGTTTTTTT

Tyrosyl tRNA syntethase – L124

>APMV/SAMBA VIRUS (100%)
TAGAATCATATAAATTATTTGATGGCGGCTAAATTATTTTGAAGGTTGTTGGTAAGATTTGACATTTGATAACAATTCAGACAATTCTGGTTTTTTGAAGTGTTCTCTAACTAGATCAATAATAGTGTTAATATAATTGGCTACA
TCAGTTTTAAGTTCACGTTTATTCATACTGGAAAAATCTTCTTGAATTGACTCGATATCTGTGTATATTTTACCACATAAGTTTAATGTACCAAACCATCTCAAAAGCAAATATTTGATGTATTCGAAAATTGGATTGTCAAAAG
TTTCATCAGTACAATATGCTCTAGATATTTTTTCACTGACTTCTTGTTCAGTATCATCCATAAAAATTGCACCTTGAGGATCAGATTTACTCATTTTTTTTTTTTGGACCAGATAGACTCATTAACATGTGATGTGATAGAGAAAT
TGGGATTTTTAATCCACGGTCATTTGCATATTCAATAGCCAACATATTGACTTTACGTTGATCAATTCCTAGTTGACAAATGTCAATTCCTTCAGGAACTAATTCAAATACGTCTGCAGCTTGCATACAAGGATAGAAAATTTGT
GAGGCTTTTAAACAATCACTTTCATTACGACCCATGATTTGACAACATCTTTTAACTCTAGATATAGTAGAAAATTCAGCTATATCTAACATTCTCTCGATATAAGATGGATTAGAAGCAATGAATTCACTGGCCCAAATAAATC
TTGTACCATCCAAATTAATACCACATGCTTTGAAAACTTCAATAAAATATCTTCCAAGTTCTCTAATCTTATTAATATCTCCATTCATTTTCAAATTCATTTTGGCAAACCAATCGGCAATATAGATAATCATCTGTCCACCACA
TTCAATAATATTATTGGTGTTCATTACAGTAATAAGAGCTTGAGCAATATGAATACGACCACTAGGTTCAAAACCATTGTAAGCTGTAAAAATTCTTCCTGAATCAACTAATTGTTTGAGTCGATCTAATGTTTCACATTCTTCT
GCTATTGATAGAAGTTGAGTCAAACGATGTTCATTATTGGTATGGTCTGTGTTTTC**CAT**TTGGATATGTATGATTTGTGAATGAAGACGAATATAAATTTAGGACAAAAAATTATCAATTTT

>APMV M4 (deleted)

>KROON VIRUS (92% ID, 0% gap)

ATTATTTGATAACGGTTAAATTATTTTGAAGATTGTTGGTAAGATTTGATATTTGACAATAAGTCAGACAATTCTGGTTTTTTAAAGTGTTCTCTAACTAGGTCAATGATAATGTTAATATAATTGGCAACATCAGTTTTAAGTT
CACGTTTATTCATACTGGAAAAATCTTGTTCAATTGACTTAATATCTGTGTATACTTTACCACATAAGTTCAATGTACCAAACCATCTCAAAAGTAAATATTTGATGTATTCAAAAATTGGATTGTCAAAAATTTCATCAGTACA
ATATGCTCTAGATATTTTTTCATTGATTTCTTGTTCAGTATCATCCATAAAAATTGCACTTTGAGGATCGGATTTACTCATTTTTTTTTTTTGGACCATTTAGACTCATTAACATGTGATGTGACAGAGAAATTGGGATTTTTAAT
CCACGATCATTTGCATATTCAATAGCCAACATATTAACTTTTCGTTGATCAATTCCTAGTTGACAAATATCAATTCCACCAGGAACTAATTCAAATACATCAGCAGCTTGCATACAAGGATAAAAAATTTGTGAGGCTTTTAAAC
AATCACTTTCATTGCGACCCATGATCTGACAACATCTTTTAACTCTAGATAGAGTAGAAAATTCTGCTATGTCTAACATTCTCTCAATATAAGATGGATTAGAAGCAATAAATTCACTCGCCCAAATAAATTTTGTACCACCCAA
ATTAATACCACATGCTTTGAAAACTTCAATAAAATATCTTCCGAGCTCTCTAATCTTCTCAATATCTCCATTCATTTTCAAATTCATTTTGGCAAACCAATCGGCAATATAAATAATCATTTGTCCACCACATTCAATAATCTTA
TTGGTATTCATTACGGTGATGAGAGCTTGAGCAATATGAATACGACCACTTGGTTCAAAACCATTATAAGCTGTAAAAATTTTTCCTGAATCAACTAATTGTTTGAGTCGGTCTAGTGTTTCACATTCTTCGGCGATTAATAGAA
GTTGATTTAAGCGATGTTCATTGTTGGAATAATCTGTGTTTTC**CAT**CTGAATACAAATTGATAATTTTATAAATGAA-ACAAGTATAAATCAAGACAATAAAATTATCAATTTT

>0YSTER VIRUS (99% ID, 0% gap)
TAGAATCATATAAATTATTTGATGACGGCTAAATTATTTTGAAGGTTGTTGGTAGGATTTGACATTTGATAACAATTCAGACAATTCTGGTTTTTTGAAGTGTTCTCTAACTAGATCAATAATAGTGTTAATATAATTGGCTACA
TCAGTTTTAAGTTCACGTTTATTCATACTGGAAAAATCTTCTTGAATTGACTCGATATCTGTGTATATTTTACCACATAAATTTAATGTACCAAACCATCTCAAAAGCAAATATTTGATGTATTCAAAAATCGGATTGTCAAAAA
TTTCATCAGTACAATATGCTCTAGATATTTTTTCACTGACTTCTTGTTCAGTATCATCCATAAAAATTGCACCTTGAGGATCAGATTTACTCATTTTTTTTTTTTGGACCAGATAGACTCATTAACATGTGATGTGATAGAGAAAT
TGGGATTTTTAATCCACGGTCATTTGCATATTCAATAGCCAACATATTGACTTTACGTTGATCAATTCCTAGTTGACAAATGTCAATTCCTTCAGGAACTAATTCAAATACGTCTGCAGCTTGCATACAAGGATAGAAAATTTGT
GAGGCTTTTAAACAATCACTTTCATTACGACCCATGATTTGACAACATCTTTTAACTCTAGATATAGTAGAAAATTCAGCTATATCTAACATTCTCTCGATATAAGATGGATTAGAAGCAATGAATTCACTGGCCCAAATAAATC
TTGTACCATCCAAATTAATGCCACATGCTTTGAAAACTTCAATAAAATATCTTCCAAGTTCTCTAATCTTATTAATATCTCCATTCATTTTCAAATTCATTTTGGCAAACCAATCGGCAATATAGATAATCATCTGTCCACCACA
TTCAATAATCTTATTGGTGTTCATTACAGTAATAAGAGCTTGAGCAATATGAATACGACCACTAGGTTCAAAACCATTGTAAGCTGTAAAAATTCTTCCTGAATCAACTAATTGTTTGAGTCGATCTAATGTTTCACATTCTTCT
GCTATTGATAAAAGTTGATTCAAACGATGTTCATTATTGGTATAGTCTGTGTTTTC**CAT**TTGGATATGTATGATTTGTGAATGAAGACGAATATAAATTTAGGACAAAAAATTATCAATTTT

Cysteinyl tRNA synthetase – L164

>APMV/SAMBA VIRUS/APMV M4 (100%)

TGCTATTATTACCTTTATTATTGTTTAATAAAATCAATGTTTTTGACAGTTATTAACTATAGTGATGTAGTGTGTATTAAGTAGTAGAGTGTATTACTCAGACGATTGAACACAAGAATTCTCGTACCACAAACTAGAATCTTTG
GAATCTTCCAAAATAATACCTATGTCGGGTAATTGAATATTACGTTGACGATCCAAAATATCAAAAAGTTGCTTTTTAATTCCAGGAGAAAGATCCGGATTTCTTGTCAATTGTCTTAATTGAGAGCGAGTCTCAATCAGAATAT
TCATGAGATCTTTAATTTTATGGGAACTACTACTATTTCCCACCCTATAAATAAAACCTAATTTGTCTAATAAATCCAGTAAATAATCACGAATTTTACCAACAATAGATTCATTTGGTCTGGGCAAATCTAAATAAACATTTGT
TGTGCCAATTAAATGCTGAATAGATCTAGCAACCATTTCAAATTTAAATTCAGTCAAATAACTGTAAATACGTTGTTGAATTCGATAAAAATCATCATGTAGCAAAGTTTCTTTGTCATTGAATTCAACATCAGATACTTCAAAT
GGATAATTGGAAACTCTATTGACAAAATTAACTACTGTAAAATCTAATTCTTTTGCGATAGAGATTAATCCATCAGTAAAATCAACTTGTTGTTTCCATTTTGTTGACATGAATAACCATCTAAATTGATTTGAATTAATTTTGT
CAAGCATTTCTTTAATGGTTGAAAAAATTCTTTAGAGACTTTGACATTTTCTGACCCTTGATACAAAGATGTCCAACATGAATAAACTCTCTAGTCCACGTATGAAAAATCATTGTGTCACTTTGATGGAGAGGATTATACATTGG
ATGATGATAAGCATTAGCCTGTAAACATTCATTATAATGATGAGGAAATTTCAAATCGATTCCACCAAAATGAATATCAATACTATTTCCTAATGTTTTCTTGATCATGGCTGAACATTCAATATGCCAACCAGGTACACCAAAT
GATTTAAATGTTTGGTTATCAAAAATAAATTCCACATTAAATCCTACATCGGATTCTGATCGACCTTTCCAAAGAGCAAAATCTTTATGGTGTTTTTTTTGGGAAACTATTTCTTTAGAAAGAAGAGATTCGTACTGTTGTTCTT
CTTCATCGTCAATTTCACTAAATTCATATCCTGCTTTTTTGTATTCAATTGAGTCAAAATAAACCGAACTATCTGATACAATATAAGCAAAACCGTTATTAATAATCTGTTGGATATATAACACAATATCACTGATGGATTCTGT
TACTCTAATCACAGAATCTGGTCTTGTGACATTTAATTTAGACATACAATCAAAAAAAGAATTTTCATGGAGTCTTGCCAATTCAAGCCAAGTAATTCCTTTGTTTTTTGATTCTCGGATAATTTTATCATCAATATCTGTAACA
TTCATTACTAGATGAGTAGGTTTGTTAAGGATTTTATTCATGGTTCGATTAATTAAATCGACAATAACATAGATTCTGGCATGTCCAATATGCGCATCATTGTAAACTGTTGGACCACATACATACATTTTTGTAACAGTGGGTA
AAATAGTTTCAGATAATTCCGTCTC**CAT**GTCAATTACTGATTGTTGTCAAAATATTATTGATTATTTAATTAGTATTTTGTTATACATTTAATAATAAATTAAATATAACAAAATAATATTTCAATTTTTATTGGAATCAGATTT
TGATGTTTTACTCCTATTGTAAACAGTTTTACATTTGATACATTGATAATATAACGTATTAGTTTTGTAATGTGTCTGATATTTAATATTTTTACACACCTTACATCGATACCAAATTACTATCATTTAAGATTTGGATTTAATA
TTGAATCATTAAGTTATTAAAATAATTTTAATAACGATAT

KROON VIRUS (90% ID, 1% gap)

---TTATTAATTTATAGTGATATAGCGTATATCAAATGGCGCAAT-
ATTATTCAGACGATTGAATACAAGAATTTTCATACCATAAACTTGAATCTTTGGAATCTTCCAAAATAATACCTATGTCGGGTAATTGAATATTACGTTGACGATCCAAAATATCAAAAAGTTGCTTTTTAATTCCAGGAGAAAT
ATCTGGATTTCTTGTCAATTGTCTTAATTGAGAGCGAGTCTCAATTAGAATATTCATGAGGTCTTTAATTTTATGGAAACTACTACTATTTCCCATTTTATAAACAAAACCTAATTTGTCTAATAAATCCAGTAAATAATCACGG
ATTTTATTAACAATAGACTCATTTGGTCTGGGTAAATCTAAATAAACATTTGTTGTCCCAATTAAATGCTGAACAGATCTAACAACCATTTCGAATTTAAATTCAGTCAAATAACTGTAAATACGTTGTTGAATTCGATAAAAAT
CATCATGTAGTAAAGTTTCTTTGTCGTTAAATTCAACATCTGATACTTCAAATGGATAATTGGAAACTCTATTAACAAAATTAACGACTGTGGAATCTAATTCTTTTGCGGTTGAAATTAATCCATCGGTAAAATCAACTTGTTG
TTTCCATTTTGTTGACATAAATAACCATCTAAACTGATTAGAATTAATTTTGTCAAGCATTTCTTTGATGGTTGAAAAAATTCTTTAATGACTTTGACATTTTCTGACCTTTGATACAAAGATGTCCAACATGAATAAACTCTCTA
GTCCACTTATAAAAAATCATTGTGTTACTTTGATGAAGAGGATTATACATTGGATGATGATAAGCATTAGCTTGTAAACATTCATTATAATGGTGAGGAAATTTCAAATCAATTCCACCGAAATGAATATCGATACTATTTCCTA
ATGTTTTCTTGATCATGGCTGAACACTCAATATGCCAACCAGGTACACCAAATGATTTAAATGTTTGGTTATCAAAAACAAATTCCACATTAAATCCTACATCAGATTCTGATCGACCTTTCCAAAGAGCAAAATCTTTATGATG
TTTCTTTTGGGAAACTATTTCCTTTGAAAGAAGAGATTCATATTGTTGTTCTTCTTCATCGTCAATTTCACTAAATTCATAACCAGCTTTTTTGTATTCAATTGAGTCAAAATACACTGAACCATCTGATACAATATAAGCAAAA
CCATTATTAATAATCTGTTGGATATATAACACAATATCACTAATAGATTCTGTTACTCTAATCACAGAATCTGGTCTCGCAACATTTAATTTAGACATACAGTCAAAAAAAGAATTTTCATGGAGTCTTGCTAGTTCGAGCCAAG
TAATTCCTCTATTTTTTGATTCTCGAATAATTTTATCATCTATATCTGTAATATTCATTACAAGATGAGTAGGTTTATCGAGGATTTTATTCATAGTTCGATTAATTAAATCGACAATGACATAGATTCTGGCATGTCCAATATG
CGCATCATTGTAAACTGTTGGACCACATACATACATTTTTGTAACAGTTGGTAAATTAGTTTTGGATAATTTTGTGTC**CAT**GTTGATTATTGATTGTTATTAAAATATTATTAAATATCAATGTAGTATTTTGTTATTTATGTAG
TATTTTGTTATTTATGTGATATTTTGTTAAATATTAGATAAATAATATTTCAATTTTTATTTAAACATAATTGGATTTCAATGTTCCAGTTCTATCATATGTGGTTTTACACTTGGTACATTGATAGTATAACATATTAGTTTTA
CGATGTATTTGATATTTAATATTCTTACATATTATACATCGATACCAAATTA-TATCA--

>OYSTER VIRUS (98%ID, 0% gap)

TGCTATTATTATCTTTATTATTGTTTAATAAAATCAATGTTTTTGACAGTTATTAACTATAGTGATGTAGTGTGTATTAAGTAGTAGAGTGTATTACTCAGACGATTGAACACAAGAATTCTCGTACCACAAACTAGAATCTTTG
GAATCTTCCAAAATAATACCTATGTCGGGTAATTGAATATTACGTTGACGATCCAAAATATCAAAAAGTTGCTTTTTAATTCCAGGAGAAAGATCCGGATTTCTTGTCAATTGTCTTAATTGAGAGCGAGTCTCAATCAGAATAT
TCATGAGATCTTTAATTTTATGGGAACTACTACTATTTCCCACCCTATAAATAAAACCTAATTTGTCTAATAAATCCAGTAAATAATCACGAATTTTACCAACAATAGATTCATTTGGTCTGGGCAAATCTAAATAAACATTTGT
TGTGCCAATTAAATGCTGAACAGATCTAGCAACCATTTCAAATTTAAATTCAGTCAAATAACTGTAAATACGTTGTTGAATTCGATAAAAATCATCATGTAGCAAAGTTTCTTTGTCATTGAATTCAACATCAGATACTTCAAAT
GGATAATTGGAAACTCTATTGACAAAATTAACTACTGTAGAATCTAATTCTTTTGCAATAGAGATTAATCCATCAGTAAAATCAACTTGTTGTTTCCATTTTGTTGACATGAATAACCATCTAAATTGATTTGAATTAATTTTGT
CAAGCATTTCTTTAATGGTTGAAAAAATTCTTTAGAGACTTTGACATTTTCTGACCCTTGATACAAAGATGTCCAACATGAATAAATTCTCTAGTCCATTTATGAAAAATCATTGTGTCACTTTGATGAAGAGGATTATACATTGG
ATGATGATAAGCATTAGCCTGTAAACATTCGTTATAATGATGAGGAAATTTCAAATCGATTCCACCAAAATGAATATCAATACTATTTCCTAATGTTTTCTTGATCATGGCTGAGCATTCAATATGCCAACCAGGTACACCAAAT
GATTTTAGTGTTTGGTTATCAAAAATAAATTCCACATTAAATCCTACATCGGATTCTGATCGACCTTTCCAAAGTGCAAAATCTTTATGATGTTTCTTTTGGGAAATTATTTCTTTGGAAAGAAAAGATTCGTACTGTTGTTCTT
CTTCGTCGTCAATTTCACTGAATTCATATCCTGCTTTTTTGTATTCAATTGAGTCAAAATAAACCGAACCATCTGATACAATATAAGCAAAACCGTTATTAATAATCTGTTGGATATACAACACAATATCACTAATAGATTCTGT
CACTCTAATCACAGAATCTGGTCTTGTGACATTTAATTTAGACATGCAATCAAAAAAAGAATTTTCATGGAGTCTTGCTAATTCGAGCCAAGTAATTCCTTTGTTTTTTGATTCTCGAATAATTTTATCATCAATATCTGTAACA

TTCATTACTAAATGAGTGGGTTTGTTAAGGATTTTATTCATGGTTCGATTAATTAAATCGACAATAACATAGATTCTAGCATGTCCAATATGAGCATCATTGTAAACTGTTGGACCACATACATACATTTTTGTAATTAGTGGGTA
AAATAGTTTCAGATAATTCCGTCTC**CAT**GTCAATTACTGATTGTTGTCAAAATATTATTGATTATTTAATTAGTATTTTGTTATACATTTAATAATAAATTAAATATAACAAAATAATATTTCAATTTTTATTGGAATCAGATTT
TGATGTTTTACTCCTATTGTAAACAGTTTTACATTTGATACATTGATAATATAACGTATTAGTTTTGTAATGTGTCTGATATTTAATATTTTTACACACCTTACATCGATACCAAATTACAATCATTTAAGATTTGGATTTAATA
TTGAATCATTGAGTTATTAAAATAATTTTAATAACGATAT

Methionyl tRNA synthetase –R639

>APMV/SAMBA VIRUS/APMV M4 (100%)

AAAATTGAAAAATTTATTGACAAATATACATATGATCCATATAAATACCTAGATTAAATATCATTA**ATG**CAAAAATTCTTTGTTACATCAGCTCTTCCTTATCCCAACAATTCATCACCACATTTAGGTAATCTTGTTGGTGCTT
TATTAAGTGGTGATGTTTATGCTCGATTTAAGCGTAATCAAGGTCATGAAGTAATCTATTTGTGTGGTACTGATGAATATGGAACTACAACAATGATTAGAGCTCGTAAGGAAGGTGTAACTTGTCGTGAACTTTGTGATAAGTA
TTTTGAGTTACACAAAAAAGTTTATGATTGGTTTAATATTGAATTTGATGTTTTTGGTAGAACATCTACAACAAAACAGACTGAAATTACTTGGGAAATATTCAATGGACTCTATAATAATGGGTACATTGAAGAAAAAACCACC
GTTCAAGCATTTTGTGAAAAATGTGATATGTATTTAGCGGATACATATCTTAAAGGATATTGTTATCATGATGGATGTCGTGAAAATAGAGTAATTTCAAATGGTGATCAGTGTGAAATTTGTCAAAAAATGATAGATGTTAATA
AACTCATAAATCCATTTTGTAGTATTTGTTTAACTCCACCCATTCAAAAATCAACTGATCATTTATATTTGTCTCTAGATAAATTAACTCCACTCGTTCAACAATATCTTGATAGAGTAGAATTTGATTCTAGAATAATGGCTAT
TTCAAAAGCTTGGTTAGAAATTGGTTTAAATCCGCGATGTATTACTAGAGATTTAGAATGGGGTACTCCAATTCCCATCAATTTGGATCCCAAACTAGAAAAATATGCTGATAAAGTTTTTTATGTATGGTTTGATGCTCCTATT
GGATACTATTCTATTTTGGCAAATGAACGTGATGATTGGCGTGAATGGCTGAATTCAGGTGTAACTTGGGTATCAACTCAAGCAAAAGATAATGTACCATTTCATTCAATAGTTTTTCCGGCTAGTGTGATTGGTTCTAATATTG
AATTACCATTGATTGACAGAATTTGTGGAACAGATTATCTACTTTATGAAGGTCAAAAATTTTCCAAAAGTCAAGGAGTTGGATTGTTCGGAGACAAAGTCGCTGAAATTTCCCCAAAATTGGGAATTAATGAAGATTATTGGAG
ATTTTATTTAATGAAAATTCGTCCTGAAACTCAAGATTCTTCATTTAATCTAGAAGAATTTGTTAGAATAGTGAAAACTGATCTTGTGAATAATATAGGTAATTTTATTAACAGAGTATTTAGTTTGCTCGAAAAAACTCCTTAT
AGAGATTTGAATTATCAAATTAGTCCGGAATATATTGAATTTATTAAAAAAATATGAAGTATCTATGGATGAATTCAAATTTAGAGATGGTTTGAAAATTTGTCTAGAAATGAGTTCCAGAGGTAATAAATTCGTTCAATCTACAA
AACCTTGGACCATGATTAAAGATGGATTGGATACACAAGAAATTATGACAGAAGCTGTTGGTATCTGTTGGATTTTGTTGAATTTATTGAAACCAATTATTCCGAAATCAGCGTGTGATATGTTGTCTAATTTAGACACTGATAA
TCAAAATATATTTTGTCTAATTGGTGGATCCAACATTAATATTAGGATACTTAATATCATTAAATTACCTTTTAAAAAATATTGATCTCAAACAACTTCGCGAATTTATTGAAGGTAAAAACTAGTCTTCTATTAGCACAAACGCA
TAAAATCAAATTAATATTATCTAATAGTTAAATACTATTAATCTGAT

>KROON VIRUS (95% ID, 0% gap)

ATCCAAATTAAATATCCAAATTAAATATCATTG**ATG**CAAAAATTCTTTGTTACGTCAGCTCTTCCTTATCCTAACAATTCATCACCACATTTAGGTAATCTTATTGGTGCTTTATTGAGTGGTGATGTTTATTCTCGATTCAAGC
GTAACCAAGGTCATGAAGTAATCTATTTGTGTGGCACTGATGAATATGGTACTACAACAATGATTAGAGCTCGTAAGGAAGGTGTAACTTGTCGCGAACTTTGTGATAAGTATTTTGAGTTACACAAAAAAGTTTATGATTGGTT
CAATATTGAATTTGATGTATTTGGTAGAACATCTACATCAAAACAGACTGAGATTACTTGGGAAATATTCAATGGACTCTATAATAATGGGTATATTGAAGAAAAAACCACTGTTCAAGCATTTTGTGAAAAATGCGACATGTAT
TTAGCAGATACATATCTTAAAGGATATTGTTATCATGATGGATGCCGTGAAAATAGAGTAATTTCAAATGGTGATCAATGTGAAATTTGTCAAAAAATGATAGATGTTAATAAACTTATAAATCCATTTTGTAGTATTTGTTTAA
CTCCACCTATTCAAAAATCAACTGATCATTTATATTTGTCTCTAGATAAATTAACTCCACTTGTCCAACAATATCTTGATAGAGTTGAATTTGATTCTAGAATAATGGCTATTTCAAAAGCTTGGTTAGAAATTGGCTTGAATCC
ACGATGTATTACTAGAGATTTAGAATGGGGTACTCCGATTCCCATCAAATTAGATCCCAAACTAGAAAAATATGCTGATAAAGTTTTTTATGTATGGTTTGATGCTCCTATTGGATACTATTCTATTTTGGCGAATGAACGTGAT
GATTGGCGTGAATGGTTGAATTCAGGTGTAACTTGGATATCAACTCAAGCAAAAGATAATGTACCATTTCATTCAATAGTTTTTCCAGCTAGTGTTATTGGTTCTAATATTGAATTACCATTGATTGATAGAATTTGTGGAACAG
ATTATCTACTTTATGAGGGTCAAAAATTTTCTAAAAGTCAAGGAGTTGGATTATTCGGAGATAAAGTTGCCGAAATTTCTCCAAAAATGGGTATTAATGAAGATTATTGGAGATTTTACCTAATAAAAATTCGTCCTGAAACTCA
AGATTCTTCATTTAATCTAGAAGAATTTGTCAGAATAGTAAAAACTGATCTTGTAAACAACATAGGTAATTTTATTAATAGAGTATTTAGTTTGCTCGAAAAAACTCCTTATAGAGATTTGAACTATCAAATTAGTCCGGAATAT
ATTGAATTTATTAAAAAATATGAAGTGTCAATGGATGAATTCAAATTTAGAGATAGTTTGAAAATTTGTCTAGAAATGAGTTCTAGAGGCAATAAATTCGTTCAATCTACAAAACCTTGGACTATGATTAAAGATGGATTGGATA
CACGGGAAATTATGACAGAAGCTGTTGGAATCTGCTGGATATTATTGAATTTATTAAAACCAATTATTCCGAAATCAGCATGTGATATGTTGTCTAATTTAGATACTGATAATCAAAATATATTTTGTCTAATTGGTGGATCTAA
CATTAATATTAGAATACTTGATATTATTAAATTACCTTTTAAAAAATATTGATCTCAAACAACTTCGTGAATTCATTGAAGGTAAAAATTAGTCTTTTATTAACACAA-
CGCACCAAAATCAAATTAATATTATCTAATAGTTAAATAATATTAATCTGAT

>OYSTER VIRUS (99% ID, o% gap)

AAAATTGAAAAATTTATTGACAAATATACATATGATCCATATAAATACCTAGATTAAATATCATTA**ATG**CAAAAATTCTTTGTTACATCAGCTCTTCCTTATCCCAACAATTCATCACCACATTTAGGTAATCTTGTTGGTGCTT
TATTAAGTGGTGATGTTTATGCTCGATTTAAGCGTAATCAAGGTCATGAAGTAATCTATTTGTGTGGTACTGATGAATATGGAACTACAACAATGATTAGAGCTCGTAAGGAAGGTGTAACTTGTCGTGAACTTTGTGATAAGTA
TTTTGAGTTACACAAAAAAGTTTATGATTGGTTTAATATTGAATTTGATGTTTTTGGTAGAACATCTACAACAAAACAGACTGAAATTACTTGGGAAATATTCAATGGACTCTATAATAATGGGTACATTGAAGAAAAAACCACC
GTTCAAGCATTTTGTGAAAAATGTGATATGTATTTAGCGGATACATATCTTAAAGGATATTGTTATCATGATGGATGTCGTGAAAATAGAGTAATTTCAAATGGTGATCAGTGTGAAATTTGTCAAAAAATGATAGATGTTAATA

AACTCATAAATCCATTTTGTAGTATTTGTTTAACTCCACCCATTCAAAAATCAACTGATCATTTATATTTGTCTCTAGATAAATTAACTCCACTCGTTCAACAATATCTTGATAGAGTAGAATTTGATTCTAGAATAATGGCTAT
TTCAAAAGCTTGGTTAGAAATTGGTTTAAATCCGCGATGTATTACTAGAGATTTAGAATGGGGTACTCCAATTCCCATCAATTTGGATCCCAAACTAGAAAAATATGCTGATAAAGTTTTTTATGTATGGTTTGATGCTCCTATT
GGATACTATTCTATTTTGGCAAATGAACGTGATGATTGGCGTGAATGGCTGAATTCAGGTGTAACTTGGGTATCAACTCAAGCAAAAGATAATGTACCATTTCATTCAATAGTTTTTCCGGCTAGTGTAATTGGTTCTAATATTG
AATTACCATTGATTGACAGAATTTGTGGAACAGATTATCTACTTTATGAAGGTCAAAAATTTTCCAAAAGTCAAGGAGTTGGATTGTTCGGAGACAAAGTCGCTGAAATTTCCCCAAAATTGGGAATTAACGAAGATTATTGGAG
ATTTTATTTAATGAAAATTCGTCCTGAAACTCAAGACTCTTCATTTAATCTAGAAGAATTTGTTAGAATAGTGAAAACTGATCTTGTGAATAATATAGGTAATTTTATTAACAGAGTATTTAGTTTGCTCGAAAAAACTCATTAT
AGAGATTTGAATTATCAAATTAGTCCGGAATATATTGAATTTATTAAAAAATATGAAGTATATATGGATGAATTCAAATTTAGAGATGGTTTGAAAATTTGTCTAGAAATGAGTTCCAGAGGTAATAAATTCGTTCAATCTACAA
AACCTTGGACCATGATTAAAGATGGATTGGATACACAAGAAATTATGACAGAAGCTGTTGGTATCTGTTGGATTTTGTTGAATTTATTGAAACCAATTATTCCGAAATCAGTGTGTGATATGTTGTCTAATTTAGACACTGATAA
TCAAAATATATTTTGTCTAATTGGTGGATCCAACATTAATATTAGGATACTTAATATCATTAAATTACCTTTTAAAAATATTGATCTCAAACAACTTCGCGAATTTATTGAAGGTAAAAACTAGTCTTCTATTAGCACAAACGCA
TAAAATCAAATTAATATTATCTAATAGTTAAATACTATTAATCTGAT

Arginyl tRNA Synthetase – R663

>APMV/SAMBA VIRUS/APMV M4 (100%)

TCAATTTTTTTAATTAAAAAAAATTGATAAGTGTAATATATTAGTATATTTTATACACATTAAATTTACCATCATAGTGTAAG**ATG**CAAGATAATTTAATTTATTTGGCAAATTGTTTCCTTAATGAAGCAATTAAAACAACTTT
ACAGAATTTAAATAAGGTTAACATTATAGACACTCCTGAATTATACAGTTTTGTTAAGGGAATTAATACTGATTATCAATTCAATAAATCAACTAAATTGGCAAATGATTGTAATCTTGATAAAGAAAAAATTGTCAACGAATTG
ATAACTCAACTGAAATCAAATTCATTTTTCGAAAATATTTCTAGTGTGGAATTAGAACAAAATAAGTCGGTCAAAATTAATGGTAAGAAAACTAATACTGTTATCAAGCAAATTATGATAACATTAAATATATCAAAATTATATT
TGTCAAATAGAATTAATTTATTGTACAAAAGAATTCTTTCGGGTTCTAGTATTTATGTTCCAAATACTATTACAAAGAAAATTATTGTCGATTATTCTTCTCCAAATATTGCTAAAGAAATGCATATTGGACATTTAAGATCAAC
TATTATTGGTGAATCTATTTGTAGGGTATTAGAAATGTGTGGACACGATGTTTATCGTATTAATCATGTGGGTGATTGGGGAACTCAATTCGGTATGTTAATTGCTTATATTAAAAATAATCAAATAGAATCTTACACCATTAGT
GAACTCATGAACATTTACAAAGAATCAAGGAAATTATTTGAATCAAGTATTGATTTTAAAAACCAATCCCGATTGGAGACCGTATCATTACAAAATGGTAATATTGAAAGTATTACTATTTGGCAAAAAATTCACAAAATATCGA
TGAACTCATTTCATGAGATCTACAGTCTTCTCGGAATAAATAATCTGATTACAAAAGGAGAATCTTTTTATCAAGATCAAATGACTGAATTAGTAAATAGTTTGACTTCGGACAACAAAATCACTGTTGAAAATGACATGAAATT
AATGTTTGTCGAAGGAATTTCCAAACCGTTTATTTTACAAAAATCTGATGGAGGATTTACTTATGACACGTCAGATTTGACTGCCTTGAAATATCGTTTATTTATAGAAAAGGCTGACCATATAATATATGTTGTAGATTCCAGT
CAACAAGAACATTTTAGTCAAATGTTTCAAATTGCTGAAAAATTAGATTGGATAAAAAATCAACAACTCCAACATATTGGATTTGGTTTAGTATTGGGATCCGATGGTTCCAAATTAAAAACTCGTTCTGGAGAAACCATTAAAT
TACAAGATGTTATTGATAATGTTGTTTCTCATGCATCTAATATTACTCGAGAATTAATCAAACAAAAAAATCTTGATTGGAATGATGATGATATTTTGACTATTTCCAAGAAAATAGCAATTAATTGCATTAAATATTCGGATCT
AAATAATCCTAGACTAAACAATTACAAATTTGATATCAATAAAATGCTTAATTCAAAAGGTAATACAGCTGTATATCTAATGTATGGATTAGCTCGTTGTAAAAGTATTTTAAGGAAAGTTCCAAATAATACTGTTTTGAATGGT
GATATTATTATTGAAAATGAAAATTCCAGGAATTTATTACTACATGTTCTAAAATATGTTGAAGTGATTGATCAAACTGTCGAAACAATGTGCCCACATTATCTTTGTATTTATTTGTATGATTTGATTGGATCTCTTACTAAAT
TTTATACAACAAATAGGTGTTTGGAATATGATAATGATAATTTAATTGGATATAATGCAAATAATTTACGCATAGTAAATATGGTCAAAATAATAATTTCCAAAATATTCGAATTGATTGGTTTAGAAGAAATTGAACAGTTATA
AATAACTTTTTGTCATGTCGATAAACATGGCAAAAGAGTATAATGTCTGTCATAAAAAATTGAAATTATTTTTGTAGATTTAATCAATTCATTAAATCCACAAAAATAAT

>KROON VIRUS (92% ID, 0% gap)

\-------------
TTAATAAAAATTGATAAACGTAATATATTAGTATATTTTATACACATTAAAATTATTATCACAGATAAAG**ATG**CAAGATAATTTAATTTATTTGGCAAATTGTTTCCTCAATGAGGCAATTAAAACAACTTTGCAGAATTTAAAT
AAAGTTAATTATTATAGACACACCAGAATTATACAGTTTTGTTAAAGGAGTTAATACGGATTATCAATTCAATAAATCAACTAAATTGGCAAATGATTGTGATATTGATAAAGAAAAATTTGTTAACGAATTGATAATTCAACTGA
AATTGAATTCATTTTTCGAAAATATTTCTAGTCTTGAATTAGAACAAAATAAGTCAGTAAAAATTAATGGAAAAAAAACTAATACTGTTATTAAACAAATTATGATTACACTAAATATATCAAAATTATATTTGTCAAATAGGAT
TAATTTATTGTACAAGAGAATTCTCTCAGGTTTTAGTATTTATGTTCCAAATGTTGTCGCAAAAAAATTTATTGTGGATTATTCTTCCCCAAATATTGCCAAAGAAATGCATATTGGACATTTAAGATCGACTATTATTGGTGAA
TCTATTTGTAGAGTACTAGAATTATGTGGACACAATGTTTATCGGATCAATCATGTGGGTGATTGGGGAACACAATTTGGTATGTTAATTGCTTATATTAAAAATAACAAAATAGAATCTTACACCATTAGTGAACTCATGAACA
TTTATAAAGAATCAAGGAAATTATTTGAGTCAAATATTGATTTTAAAAACCAAGCACGATTGGAGACTGTATCATTACAAAATGGTAATATTGAAAGTATTACTATTTGGAAAAAAATTCACGAAATATCCATGAATTCATTTTA
TGAGATCTACAAACTTCTTGGAATAAATAATCTAGTTACAAAAGGAGAATCTTTTTATCAAGATCAAATGATCGAATTAGTAAATAGTTTAACTTTGGAAAACAAAATCACTGTTGAAAATGACATGAAATTAATGTTTATTGAA
GGAATTTCCAAACCATTTATTTTACAAAAATCTGATGGAGGATTCACTTATGATACATCAGATTTGGCTGCCTTAAAATATCGTTTATTTATAGAAAAAGCCGATCGTATAATATATGTTGTAGATTCTAGTCAACAAGAACATT
TTAGTCAAATGTTTCAAATTGCTGAAAAATTAGATTGGATAAAAAATCAACAACTTCAACATATTGGATTTGGTTTAGTATTGGGATCTGATGGTTCCAAATTAAAAACTCGTTCCGGAGAAACCATTAAACTACAAGATGTTAT
TGATGATGTTGTTTCTCATGCATCTAATATTACTCGAGAATTAGTCAAACAAAAAGATCTTGATTGGAATGATGATGATATTTTGGTTATTTCCAAGAAAATTGCCATTAATTGCATTAAATATTCGGACCTAAATAATCCCAGA
TTAAATAATTACAAGTTTGATATCAATAAAATGCTTAATTCAAAAGGTAATACAGCTGTATATTTAATGTATGGATTAGCTCGCTGTAAAAGTATTTTAAGGAAAGTTCCAGATAATACTATTTTGAATGGTGATATTATTATTG

```
AAAATGAAAATGCTAGAAATTTATTGTTACATGTGCTAAAATATGTGGAAGTGATTGATCAAACTATCGAAACAATGTGTCCACATTATCTTTGTATTTATTTGTATGATTTGGTTGGATCTCTCACTAAATTTTATACAACAAA
TAGATGTTTGGAATATGATAATAGCGATTTAATTGGATATAATACAAATAATTTACGCATAATAAATATTGTCAAAACAATAATTTCCAAAATATTTGAATTGATTGGTTTAGAAGAAATTGAGCAGTTATAAATACTTGTTT--
-------------------------------------------------------------------------------------------------
```

> OYSTER VIRUS (99% ID, 0% gap)

TCAATTTTTTTAATTAAAAAAAATTGATAAGTGTAATATATTAGTATATTTTATACACATTAAATTTAC**CAT**CATAGTGTAAG**ATG**CAAGATAATTTAATTTATTTGGCAAATTGTTTCCTTAATGAAGCAATTAAAACAACTTT
ACAGAATTTAAATAAGGTTAACATTATAGACACTCCTGAATTATACAGTTTTGTTAAGGGAATTAATACTGATTATCAATTCAATAAATCAACTAAATTGGCAAATGATTGTAATCTTGATAAAGAAAAAATTGTCAACGAATTG
ATAACTCAACTGAAATCAAATTCATTTTTCGAAAATATTTCTAGTGTGGAATTAGAACAAAATAAGTCGGTCAAAATTAATGGTAAGAAAACTAATACTGTTATCAAGCAAATTATGATAACATTAAATATATCAAAATTATATT
TGTCAAATAGAATTAATTTATTGTACAAAAGAATTCTTTCGGGTTCTAGTATTTATGTTCCAAATACTATTACAAAGAAAATTATTGTCGATTATTCTTCTCCAAATATTGCTAAAGAAATGCATATTGGACATTTAAGATCAAC
TATTATTGGTGAATCTATTTGTAGGGTATTAGAAATGTGTGGACACGATGTTTATCGTATTAATCATGTGGGTGATTGGGGAACTCAATTCGGTATGTTAATTGCTTATATTAAAAATAATCAAATAGAATCTTACACCATTAGT
GAACTCATGAACATTTACAAAGAATCAAGGAAATTATTTGAATCAAGTATTGATTTTAAAAACCAATCCCGATTGGAGACCGTATCATTACAAAATGGTAATATTGAAAGTATTACTATTTGGCAAAAAATTCACAAAATATCGA
TGAACTCATTTCATGAGATCTACAGTCTTCTCGGAATAAATAATCTGATTACAAAAGGAGAATCTTTTTATCAAGATCAAATGACTGAATTAGTAAATAGTTTGACTTCGGACAACAAAATCACTGTTGAAAATGACATGAAATT
AATGTTTGTCGAAGGAATTTCCAAACCGTTTATTTTACAAAAATCTGATGGAGGATTTACTTATGACACGTCAGATTTGACTGCCTTGAAATATCGTTTATTTATAGAAAAGGCTGACCATATAATATATGTTGTAGATTCCAGT
CAACAAGAACATTTTAGTCAAATGTTTCAAATTGCTGAAAAATTAGATTGGATAAAAAATCAACAACTCCAACATATTGGATTTGGTTTAGTATTGGGATCCGATGGTTCCAAATTAAAAACTCGTTCTGGAGAAACCATTAAAT
TACAAGATGTTATTGATAATGTTGTTTCTCATGCATCTAATATTACTCGAGAATTAATCAAACAAAAAAATCTTGATTGGAATGATGATGATATTTTGACTATTTCCAAGAAAATAGCAATTAATTGCATTAAATATTCGGATCT
AAATAATCCTAGACTAAACAATTACAAATTTGATATCAATAAAATGCTTAATTCAAAAGGTAATACAGCTGTATATCTAATGTATGGATTAGCTCGTTGTAAAAGTATTTTAAGGAAAGTTCCAAATAATACTGTTTTGAATGGT
GATATTATTATTGAAAATGAAAATTCCAGGAATTTATTACTACATGTTCTAAAATATGTTGAAGTGATTGATCAAACTGTCGAAACAATGTGCCCACATTATCTTTGTATTTATTTGTATGATTTGATTGGATCTCTTACTAAAT
TTTATACAACAAATAGGTGTTTGGAATATGATAATGATAATTTAATTGGATATAATGCAAATAATTTACGCATAGTAAATATGGTCAAAATAATAATTTCCAAAATATTCGAATTGATTGGTTTAGAAGAAATTGAACAGTTATA
AATAACTTTTTGTCATGTCGATAAACATGGCAAAAGAGTATAATGTCTGTCATAAAAAATTGAAATTATTTTTGTAGATTTAATCAATTCATTAAATCCACAAAAATAAT
